# Malocclusion Complexity in Patients with Dental Anomalies—A Case–Control Study

**DOI:** 10.3390/dj13110506

**Published:** 2025-11-03

**Authors:** María Fernanda Romero-Noh, José Rubén Herrera-Atoche, Iván Daniel Zúñiga-Herrera, Bertha Arelly Carrillo-Ávila, Víctor Manuel Martínez-Aguilar, Laura Beatriz Pérez-Traconis

**Affiliations:** School of Dentistry, Autonomous University of Yucatan, Mérida 97000, Mexico; a15001966@alumnos.uady.mx (M.F.R.-N.); ivan.zuniga@correo.uady.mx (I.D.Z.-H.); arelly.carrillo@correo.uady.mx (B.A.C.-Á.); victor.martinez@correo.uady.mx (V.M.M.-A.); laurap@correo.uady.mx (L.B.P.-T.)

**Keywords:** dental occlusion, malocclusion, tooth abnormalities, tooth agenesis, tooth impacted

## Abstract

**Background/Objectives**: This study aimed to evaluate the impacts of various dental anomalies on the complexity of malocclusion. **Methods**: This retrospective cross-sectional study employed a case–control design. The sample comprised 140 patients, 59 cases, and 81 controls. The Index of Complexity Outcome and Need (ICON) was used to calculate a score indicating the complexity of the malocclusion. According to the ICON score, the level of malocclusion complexity was classified into easy, mild, moderate, difficult, and very difficult. The cases were subdivided into three groups based on their dental anomaly type (number, shape, or eruption anomalies). A chi-square test was used to compare the distribution of cases and controls across the ICON levels (*p* < 0.05). A *t*-test and an ANOVA with Tukey’s post hoc test were used to evaluate the differences in the ICON scores among groups (*p* < 0.05). **Results**: The mean values of the ICON score were 56.77 ± 17.1 for the cases and 47.44 ± 17.54 for the controls (*p* = 0.002). Most patients in the case group were within the highest three ICON levels, while most controls were in the lowest three (*p* = 0.022). Patients with eruption anomalies had a higher ICON score, compared to the controls and those in other dental anomaly groups (*p* = 0.001). **Conclusions**: The presence of dental anomalies increases the complexity of malocclusion. Eruption anomalies are more complex to resolve than number and shape anomalies, due to their impact on occlusion and aesthetics.

## 1. Introduction

Dental anomalies refer to a group of alterations of the dentition that affect both primary and permanent teeth. According to their type, dental abnormalities are classified as number, shape (size/morphology), eruption, or structure abnormalities [1]. Their prevalence varies between different geographic regions and ethnic groups; for example, the reported prevalence in Croatia is 24.1% [2], which is one of the lowest values in comparison to other countries such as Saudi Arabia with 27.8% [3], Mexico with 28.05% [4], Egypt with 32.6% [5], India with 34.28% [6], Turkey with 40.3% [7], or France with 45.74% [8].

The most prevalent dental anomaly type also varies among populations; for example, in some countries such as Turkey [7] or Croatia [2], number anomalies are the most prevalent; meanwhile, in countries such as Mexico [4] or Egypt [5], eruption anomalies are most frequent.

Different types of dental anomalies are commonly present simultaneously in the same individual, and it has been calculated that approximately 14.16% of subjects present more than one dental anomaly simultaneously [8]. There are two reasons for this observation. One is that some dental anomalies share a common genetic origin, so it is frequent to see an eruption anomaly accompanied by a shape anomaly [4,9]. The second reason is that some anomalies are a consequence of others; for example, a central upper incisor may be blocked in its eruption path due to the presence of a mesiodens [10,11,12].

Regardless of their type, all dental anomalies affect the dental occlusion and aesthetics. For example, the unilateral agenesis of an upper lateral incisor can be a challenge to treat and may require a multidisciplinary approach to achieve optimal outcomes [13]. Therefore, dental anomalies can significantly impact the onset of dental malocclusions. Interestingly, in 2009, Uslu et al. investigated whether the presence of dental anomalies was associated with the Angle malocclusion classification in a group of Turkish patients, but they found no significant relationship [7]. More recently, Ja Hyeong Ku et al. (2020) evaluated a group of Korean patients and found a significant association between the presence of dental anomalies and the Angle malocclusion classification [14]. The differences between these studies may be due to ethnic disparities and differences in the methodology used (e.g., Uslu et al. divided the class II subjects into divisions 1 and 2) [7]. Although orthodontists widely use the Angle classification for clinical purposes, it has the downside that it only evaluates malocclusions in the sagittal dimension. In the present study, we propose using the Index of Complexity, Outcome, and Need (ICON), which assesses malocclusions in the sagittal, transverse, and vertical dimensions, while also including an aesthetic component. Furthermore, the ICON enables evaluation of the level of malocclusion complexity and the difficulty of resolving it, which, in the case of this research, may help to understand the results from a clinical perspective [15].

This study aimed to evaluate the impacts of various dental anomalies on the complexity of malocclusion.

## 2. Materials and Methods

This retrospective cross-sectional study employed a case–control design. The required sample size was estimated by considering an etiological factor previously identified in the studied population [16]. An odds ratio of 2 was considered for the cases compared with the controls. If 54% of the cases were exposed [17] and each control was matched with one case, a minimum sample size of 74 patients would provide 95% power and a 95% confidence level (α).

The sample was obtained from the database of an orthodontic clinic of a dental school. All subjects provided written consent to participate in the study. This research was approved by the University Autonomous of Yucatan’s Institutional Review Board (FODO-2021-0001). The sample included patients aged 12 years or older with permanent dentition. Patients who had undergone orthodontic or orthopedic treatment, as well as those with extensive dental restorations or those affected by syndromes or conditions such as cleft lip and palate, were excluded.

To be included in the case group, a patient had to present at least one of eight evaluated dental anomalies, including two number anomalies (agenesis and supernumerary teeth), three shape anomalies (microdontic upper lateral incisors, barrel-shaped teeth, and peg-shaped teeth), and three eruption anomalies (impacted teeth, dental transposition, and dental transmigration). It should be noted that, due to their variability [2], third molars were excluded from the study—as is the case in many papers on this topic [4,6,11,14]. Definitions for the considered dental anomalies are given in Table 1 [4]. The control group included patients without dental anomalies.

A single operator assessed the patients’ malocclusion complexity using the Index of Complexity, Outcome, and Need (ICON). To calculate the ICON score, five components are considered: (1) aesthetics (derived from the Index of Orthodontic Treatment Need); (2) upper arch crowding or spacing; (3) crossbite; (4) incisors’ vertical relationship (overbite); (5) buccal segment sagittal relationship. According to the ICON score, the malocclusion complexity was classified into 5 levels: (1) easy (score < 29); (2) mild (score 29–50); (3) moderate (score 51–63); (4) difficult (score 64–77); and (5) very difficult (score > 77) [15]. The operator employed an unblinded method when calculating the ICON score across the groups.

Given the small number of subjects affected by certain dental anomalies (for example, peg-shaped upper laterals or dental transmigration), it was decided to classify them into dental anomaly type groups for comparison with the controls. Therefore, the cases were divided into number anomalies, shape anomalies, and eruption anomalies (only cases with single anomalies were included in these groups; subjects who presented double or triple anomalies were excluded).

### 2.1. Method of Error Control

A precisely trained single operator determined the ICON value for each patient. After training, a pilot test was conducted for intra-operator evaluation, comparing blind determinations of ICON values made twice for the same patients at different times (paired *t*-test of ICON values, *p* > 0.05). An inter-operator assessment was also performed, comparing the previously mentioned ICON determinations with those made by another expert for the same patients (independent *t*-test of ICON values, *p* > 0.05).

### 2.2. Statistical Analysis

The statistical analysis was carried out using the SPSS software (version 20; IBM, Armonk, NY, USA). A chi-square test was used to compare the distributions of cases and controls across the ICON levels (*p* < 0.05). The Levene test was employed to determine the homogeneity of variance, the Kolmogorov–Smirnov test was used to verify the normality of the data, and an eta squared was used to estimate the effect size. A *t*-test was used to compare the differences in the mean ICON scores between cases and controls. An ANOVA with Tukey’s post hoc test was performed to evaluate the differences in means between various types of dental anomalies (*p* < 0.05).

## 3. Results

The final sample consisted of 140 patients, comprising 59 cases (42.24%) and 81 controls (57.85%); the mean age of the participants was 17.23 ± 4.71 years. The distribution by sex was 62.7% (n = 37) females and 37.3% (n = 22) males in the case group, while it was 61.7% (n = 50) and 38.3% (n = 31), respectively, for the controls. Among the cases, 69.49% (n = 41) had one type of dental anomaly, 27.11% (n = 16) had two, and 3.4% (n = 2) had three.

**Figure 1:**
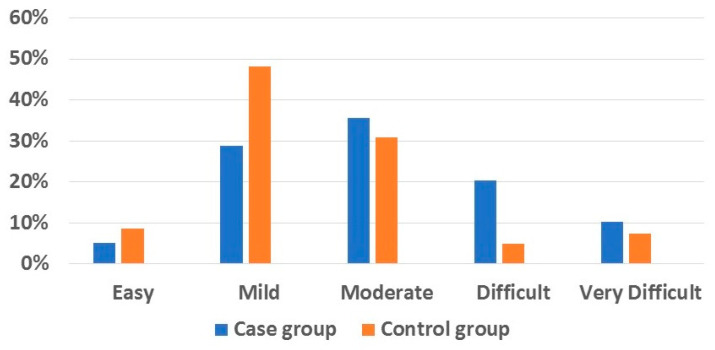
Distribution of the patients across the ICON levels.

The mean values of the ICON score were 56.77 ± 17.1 (52.32–61.23, 95% confidence interval) for the cases and 47.44 ± 17.54 (43.56–51.32, 95% confidence interval) for the controls; the difference between these values was statistically significant (*p* = 0.002), with an effect size of 0.067. Regarding the distribution of the patients across the ICON levels, most subjects in the control group were within the lowest three ICON levels. In comparison, most patients in the case group were within the highest three ICON levels (*p* = 0.022), with an effect size of 0.132 (Figure 1 and Table 2).

Finally, the ANOVA revealed that the patients with eruption anomalies had a higher ICON score compared to the controls and those in the other dental anomaly groups (*p* = 0.001) (Table 3).

## 4. Discussion

The results of this study demonstrate that the presence of dental anomalies increases the complexity of malocclusion.

Analyzing the ICON score’s components reveals that dental anomalies affect them through different mechanisms. All types of anomalies studied (i.e., number, eruption, and shape) were found to affect the aesthetic component. For example, all three types of dental anomalies could lead to a shift in the upper midline, resulting in asymmetric smiles, especially in unilateral cases [11,13,18].

Regarding crowding or spacing, all three types of dental anomaly may contribute to the development of these conditions through different mechanisms. Dental agenesis leaves edentulous spaces, while supernumerary teeth could create both crowding and spacing; in the first case by increasing the number of teeth [11]. In cases related to spacing, supernumerary teeth—such as mesiodens—are commonly associated with upper midline spacing [19]. Eruption anomalies leave spaces in the dental arch due to the lack of impacted teeth [20], while shape anomalies—such as microdontia—create spaces in the dental arch [11].

Regarding the following ICON components of crossbite and overbite, number [21], eruption [22], and shape anomalies [21] are all etiological factors for tooth size discrepancy (Bolton discrepancy). In the anterior sector of the dental arch, the presence of severe tooth size discrepancy affects the relationship between the incisors [23,24,25]. Reduced dental mass in the upper arch leads to an anterior crossbite [23], while reduced dental mass in the lower arch tends to the development of a deep bite [24].

Finally, the fifth component (buccal segment sagittal relationship) is also affected by the considered dental anomalies. The absence of a tooth creates spaces in the dental arch to which other teeth erupt [26]; this situation also applies to microdontic teeth. In some cases, this results in the molars or canines being outside the ideal Class I relationship [27]. In the case of eruption anomalies, the upper canine is the most frequently impacted tooth [2,3,4,6,7,8,28]; therefore, the sagittal relationship of the occlusion is significantly affected.

After analyzing how the considered dental anomalies may affect dental occlusion, it is interesting to explore why eruption anomalies have a more significant impact on the ICON score when compared with the other two types. The apparent explanation is that eruption anomalies must be more disruptive to occlusion and aesthetics than the other two. Additionally, these effects are more frequently observed in patients with eruption anomalies than in those with number or shape anomalies.

For this analysis, the population in which the study was conducted should be taken into consideration. The main factor to consider is: which teeth are most affected by each dental anomaly in this population? In the case of number anomalies, the second lower premolar is the most frequently absent tooth due to agenesis, while mesiodens is the most common supernumerary tooth. For eruption anomalies, the maxillary canine is the most commonly impacted tooth, followed by the upper central incisor. On the other hand, the mandibular canine is the most frequently affected tooth in dental transmigration. Finally, the three shape anomalies considered in this study all concern only the upper lateral incisors, being the teeth most commonly affected by these conditions [4].

### 4.1. Assessment of the Impact of the Presence of Dental Anomalies on the ICON’s Aesthetic and Upper Arch Crowding or Spacing Components

Considering the characteristics of the study population, eruption anomalies frequently involved absence of the maxillary canine or central incisor due to impaction [4]. In the case of the first two ICON components (aesthetic and upper arch crowding or spacing), the absence of a central incisor has a significant impact on the patient’s self-esteem and aesthetic [12,29]. Meanwhile, an impacted canine or central incisor tends to create spacing in the upper arch, especially in cases involving bilateral impactions [30]. In unilateral cases, the patient develops a shift in the upper midline [27]. Given the size of the canines compared with the size of a microdontic upper lateral, it is reasonable to think that impacted canines are more disruptive to a patient’s smile than the incisors.

Furthermore, it is worth noting that peg-shaped upper laterals were the least prevalent form of microdontic teeth in this population. The most frequent form is the upper laterals, which have a standard shape but are smaller in width than the lower lateral incisors [4]. This form is less unsightly for the smile than the peg-shaped form.

Regarding the number anomalies in this population, the most commonly absent teeth due to agenesis were in the lower arch (second premolars and lower central incisors) [4]; due to their location in the lower arch (as well as one in the posterior portion of the arch), their effect on a patient’s smile is negligible. In the case of supernumerary teeth, the mesiodens are not always erupted. It has been reported that 66% of supernumerary teeth are impacted, and only 32% of subjects affected by them presented a midline diastema [31]. Considering these prevalence values, the aesthetic of patients who present supernumerary teeth is not as frequently affected when compared to those with impacted or transposed teeth.

### 4.2. Assessment of the Impact of the Presence of Dental Anomalies on the ICON’s Crossbite and Overbite Components

In the case of the crossbite component, the two most prevalent absent teeth in this population are located in the lower arch, one of which is in the anterior zone (central incisor), predisposing to the opposite situation (increased overjet) [32]. Regarding the shape type of anomalies, there is evidence that microdontia is associated with class III malocclusion, which is often accompanied by crossbite [33].

Concerning eruption anomalies, there is controversy regarding the size of the maxillary bone and the presence of impacted teeth. Scientific evidence has demonstrated that transverse deficiency of the maxillary bone is associated with maxillary canine impaction [34,35]. However, it is essential to distinguish between buccally and palatally displaced maxillary canines. Distinct features between the two types of impacted maxillary canines have been evidenced, with buccally displaced ones being more susceptible to environmental factors such as transverse dental arch deficiencies [36]. On the other hand, palatally displaced canines are unrelated to the size of the bones [37,38], likely due to their genetic etiology [39]; thus, multiple factors contribute to the development of this condition. In any case, transverse deficiency of the bones is considered an etiological factor for canine impaction [36]; therefore, the presence of crossbite is a feature to look for in such cases, making it more complex to resolve.

Regarding the overbite ICON component, there is evidence in the scientific literature that subjects with palatal impaction of the canines tend to have a brachyfacial skeletal pattern [40] and, therefore, present a deep bite. For this ICON component, the agenesis of the lower second premolar and the central incisor is an etiological factor for a deep bite, as it is related to retroclination and extrusion of the incisors [41]. This is also associated with a tendency towards a hypodivergent growth pattern [33].

### 4.3. Assessment of the Impact of the Presence of Dental Anomalies on the ICON’s Sagittal Relationship Component

Finally, analyzing the sagittal relationship component, the absence of the lower teeth due to agenesis frequently leaves non-exfoliated second primary molars, which creates a “end on” class II molar occlusion [42]. For their part, microdontic upper laterals are associated with mesial canine migration as a consequence of the reduced dental mass in the anterior part of the upper arch [43]. However, eruption anomalies are the most disruptive in this context, severely affecting the sagittal relationship due to impaction or transposition of the upper canines [27,44].

In summary, this analysis revealed that all three types of dental anomaly impact occlusion in various ways. However, given that the maxillary canine is the most frequently affected tooth by eruption anomalies, these anomalies were found to be more disruptive to occlusion and aesthetics than the other two, thereby increasing the complexity and difficulty of resolving the patient’s malocclusion.

### 4.4. Limitations of This Study

This study has limitations that should be considered. First, as previously mentioned, the results may vary when applied to different ethnic groups (it also must be taken into consideration that all subjects came from a single institution). For example, Altug-Atac et al. (2007) found that upper lateral incisors were the most prevalent absent teeth due to dental agenesis in a Turkish population, while peg-shaped upper laterals were the most common type of microdontic teeth [45]. Therefore, the results for Turkish individuals may differ from those presented in this paper. Second, we isolated patients who were affected by a single dental anomaly. This is important to consider, as dental anomalies often present with other conditions. For example, in the study population, dental impaction was associated with microdontia, dental agenesis, supernumerary teeth, barrel-shaped upper laterals, and dental transposition. These associations are frequent and may vary among different types of dental anomalies, as well as between distinct ethnic groups [4]. Another limitation to consider is that, in this particular population, it has been shown that the prevalence of dental anomalies does not differ by sex [4]; however, not all ethnic groups are the same in this regard. For example, Saudi Arabian males have a higher prevalence of Taurodontism [3], Korean males present more frequently with supernumerary teeth [14] and, in Brazil, males have a higher prevalence of impacted teeth [33]. Therefore, it is important to consider this fact before generalizing the results. Finally, the retrospective design of this study presents its own limitations, including the process of participant selection, risk of missing data, and limitations in the generalizability of the results.

## 5. Conclusions

In conclusion, the presence of dental anomalies increases the complexity of malocclusion. Due to their impacts on occlusion and aesthetics, eruption anomalies are more complex to resolve than number and shape anomalies.

## Figures and Tables

**Table 1:** Definitions of dental anomalies [4].

Number Anomalies	
Dental agenesis	When there is no identifiable mineralization of the tooth crown on radiographic records, and no evidence of extraction.
Supernumerary teeth	A tooth is considered supernumerary when it appears in addition to the normal number of teeth.
Shape Anomalies	
Microdontic upper lateral incisors	An upper lateral incisor is considered microdontic when its mesiodistal width is equal to or smaller than that of its mandibular counterpart.
Barrel shaped upper lateral incisors	An upper lateral incisor is considered to be barrel-shaped when the operator identifies a pronounced manifestation of a thickened or elevated cingulum on the gingival aspect of its lingual surface.
Peg shaped upper lateral incisors	An upper lateral incisor is considered to be peg-shaped when its width is greatest at the cervical margin.
Eruption Anomalies	
Impacted teeth	The operator classifies a tooth as impacted when it is not expected to erupt fully into its regular functional position based on clinical and radiographic examinations.
Dental transposition	Dental transposition is defined as the “positional interchange of two adjacent teeth, or the development or eruption of a tooth in a position normally occupied by a non-adjacent tooth.”
Dental transmigration	A tooth is considered in dental transmigration when its “eruption path had been altered and the tooth had drifted to the opposite side of the arch with at least half of the crown length crossing the midline.”

**Table 2:** Distribution of the patients across the ICON levels and chi-square test.

	ICON Levels					*p*
	Easy	Mild	Moderate	Difficult	Very Difficult	
	% (N)	% (N)	% (N)	% (N)	% (N)	
**Case group**	5.1 (3)	28.8 (17)	35.6 (21)	20.3 (12)	10.2 (6)	0.022 *
**Control group**	8.6 (7)	48.1 (39)	30.9 (25)	4.9 (4)	7.4 (6)	

(*) Statistically significant (*p* < 0.05). ICON: Index of Complexity, Outcome and Need.

**Table 3:** ICON score comparison between control and case groups (number, size, and eruption anomaly groups), based on ANOVA with Tukey’s post hoc test.

	Groups				*p*
	Control	Number	Shape	Eruption	
	Mean SD (95% CI) N = 81	Mean SD (95% CI) N = 10	Mean SD (95% CI) N = 10	Mean SD (95% CI) N = 21	
**ICON Score**	47.44 ±17.54 ^a^ (43.56–51.32)	45.9 ±17.66 ^a^ (33.26–58.53)	41.9 ±12.52 ^a^ (32.93–50.86)	63.23 ±14.26 ^b^ (56.74 -69.72)	0.001 *

(*) Statistically significant (*p* < 0.05). ICON: Index of Complexity, Outcome and Need. SD: Standard Deviation. CI: Confidence Interval. Different letters represent significant differences between the values.

## Data Availability

The original contributions presented in this study are included in the article. Further inquiries can be directed to the corresponding author.

## Abbreviations

The following abbreviation is used in this manuscript:

ICON	Index of Complexity, Outcome, and Need
